# Experimental demonstration of cyclotron emissions in micro-scale graphene structures

**DOI:** 10.1038/s41598-024-64051-2

**Published:** 2024-06-16

**Authors:** Jordan Planillo, Dragoslav Grbovic, Fabio Alves

**Affiliations:** 1 Naval Postgraduate School, Monterey, CA USA; 2 Naval Air Warfare Center Weapons Division, Point Mugu, CA USA

**Keywords:** Electronic and spintronic devices, Photonic devices, Electronic devices, Physics, Nanoscience and technology, Graphene, Electronic properties and devices, Optical properties and devices

## Abstract

A solid-state implementation of a cyclotron radiation source consisting of arrays of semicircular geometries was designed, fabricated, and characterized on commercially available graphene on hBN substrates. Using a 10 µm design radius and device width, respectively, such devices were expected to emit a continuous band of radiation spanning from 3 to 6 GHz with a power 3.96 nW. A peak emission was detected at 4.15 GHz with an effective array gain of 22 dB. This is the first known experimental measurement of cyclotron radiation from a curved planar graphene geometry. With scaling, it may be possible achieve frequencies in the THz range with such a device.

## Introduction

There is a need to further increase the accessible spectrum into the THz range in a cost-effective manner. Access to this spectrum can find applications in wireless communications, non-destructive inspection (NDI), and spectroscopy. In the domain of wireless communications, the rollout of 5th generation^1^ (5G) wireless networks has shown that usable spectrum is congested and is becoming increasingly scarce. The needs for even more data capacity in future generations of wireless networks such as sixth generation^2^ (6G) and beyond require even more bandwidth and consequently, higher frequencies^3^.

With respect to NDI, THz radiation can penetrate soft materials without the hazards of ionizing radiation–unlike x-rays^4^. NDI methodologies have largely revolved around the examination of metallic samples with techniques such as eddy current testing^5^ and x-ray imaging. These methodologies have become less effective as composite materials increasingly become incorporated into structures. THz radiation offers a promising solution for composite materials NDI^6^. In addition to the NDI capabilities of THz radiation, it can also be used for the identification of chemical species via THz spectroscopy. This can especially be useful in security applications for the detection of illicit substances^7,8^, in health applications for monitoring hazardous conditions^9^, or monitoring vitals such as blood glucose levels^10^.

Achieving THz radiation in a compact solid-state form has proven difficult due to fundamental limitations in conventional materials and approaches^11^. These limitations may be overcome with a paradigm shift via materials selection and by adopting approaches from particle accelerator based light sources. This work details the design, fabrication, and characterization of a solid-state implementation of a cyclotron radiation source in graphene.

Electromagnetic radiation, in its most simple case is the radiation from an accelerated point charge, *q*. The resulting radiated electric field is given by the Lienard–Wiechart potentials ^12^:

1 $$\mathop{E}\limits^{\rightharpoonup} = \frac{q}{c}\left[ {\frac{{\hat{n} \times (\hat{n} \times \dot{\beta })}}{R}} \right]_{ret}$$

where *c* is the speed of light, $\hat{n}$ is the unit vector from the source point to the observer, *R* is the distance from the origin to the observer, and $\dot{\beta }$ is the acceleration normalized by the speed of light with the expression evaluated at the retarded time.

Consider the case of a charged particle, with mass *m*, in a magnetic field of magnitude, *B*, with a velocity, *v*. The charged particle will orbit in a plane whose normal vector coincides with the magnetic field. The orbital velocity, *ω*, and orbital radius, *r*, are given by the following relation ^13^:

2 $$\omega = \frac{v}{r} = \frac{qB}{m}.$$

In the case of *R* >  > *r* and *β* <  < 1, the resulting electric field reduces to that of the rotating electric dipole where the dipole moment is $p_{0} = \, q\, \cdot {\kern 1pt} \,r_{arc}$. The cyclotron is one such device that produces electromagnetic radiation from charges in circular orbit due to a magnetic field.

Graphene’s exceptional room temperature charge carrier transport properties such as mobility (*µ*_*n*_ = 2 × 10^5^ cm^2^/V·s^14^) and saturation velocity (*v*_*sat*_ = 4.25 × 10^7^ cm/s^15^) make it a desirable material for use in conventional electronic devices and for novel device applications. One such device application uses graphene as a solid-state implementation of particle accelerator-style radiation sources technologies which are most notably used as high-intensity broadband x-ray sources^16–20^. The stages of development for these devices range from theoretical possibilities to proof-of-concept fabrication; however, electromagnetic radiation measurements do not appear in the open literature.

One promising method for producing cyclotron-style electromagnetic radiation utilizing graphene is in the form of corrugated graphene^21^. By transferring graphene onto an etched grating substrate, the graphene conforms to the substrate and becomes a solid-state implementation of a wiggler ^22,23^. The radiated frequency and power are a function of the corrugation period and depth. In theory, this approach can reach frequencies as high as 2 THz.

A curved planar graphene sheet geometry is yet another approach that provides tangential acceleration and allows for a net displacement of charges. Rather than using an external magnetic field to provide the centripetal force that accelerates the charges, the graphene is patterned to the desired trajectory to achieve the desired acceleration.

While the graphene films grown by CVD occupy a continuous area, monolithic continuous sheets on the order of 1 cm^2^ do not currently exist as the graphene grows in grains which range in size from a few microns to tens of microns. Device performance will be hindered due to scattering at the grain boundaries ^24–26^. Additionally, the corrugations induce localized charge density variations as stresses on the lattice caused by the corrugations locally shift the Fermi level^27^, in which case, a planar geometry may be more desirable.

By explicitly patterning the trajectory, grain boundary can be circumvented if the patterned trajectory is within the size of a grain and can be further improved if a multitude of trajectories are patterned over the graphene film. This work reports on the fabrication and characterization of such planar semicircular graphene arc arrays as cyclotron radiation emitters.

**Figure 1:**
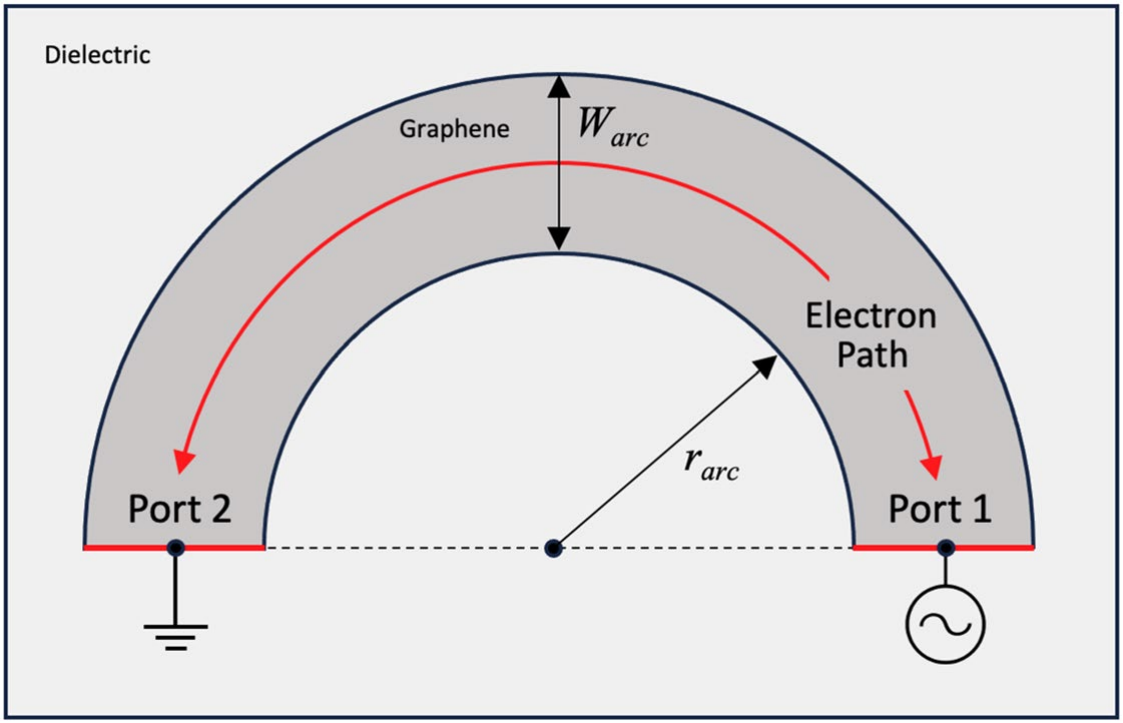
Schematic diagram of the graphene arcs. When the non-equilibrium condition is satisfied via high field bias application, the arc’s charges are concentrated at a single point and traverse a semicircular trajectory at a radius *r*_*arc*_. The motion of the charges is expected to produce cyclotron radiation at frequencies inversely proportional to *r*_*arc*_.

The device concept of operation requires that the non-equilibrium bias condition is satisfied via applied high field bias ^15,28,29^. Under this condition, the free carriers in the graphene arc will behave as a point charge, *q* ^30,31^ moving at the saturation velocity when viewed from far-field. If the graphene is patterned in a circular geometry of radius *r*_*arc*_, and width *W*_*arc*_, the effective point charge will then be constrained to move in a circular fashion (Fig. 1). Given that the saturation velocity is well within the non-relativistic regime, this geometry can be modeled as a rotating electric dipole—a well-known problem in classical electrodynamics ^12,32^. As such, the frequency emitted electromagnetic radiation, *f*_*target*_, will be proportional to the orbital velocity *ω*_*0*_ with the following relation:

3 $$f_{{{\text{target}}}} = \frac{{\omega_{0} }}{2\pi } = \frac{{v_{{{\text{sat}}}} }}{{r_{{{\text{arc}}}} }}$$

The emitted power is given by the Larmor formula:

4 $$P = \frac{{q^{2} a^{2} }}{{6\pi \epsilon_{0} c^{3} }}$$

where *q* is the effective single point charge of the graphene arc—the product of the carrier density of graphene and the arc area, *a* is the charge’s centripetal acceleration (*a* = *v*_*sat*_^2^*/r*_*arc*_), ${\epsilon }_{0}$ is the vacuum permittivity, and *c* is the speed of light in vacuum.

The resulting fields are given by:

5 $$\begin{gathered} \mathop{E}\limits^{\rightharpoonup} = \frac{{\mu_{0} p_{0} \omega }}{4\pi r}\left\{ {\cos (\theta )[\cos (\omega (t - \tfrac{r}{c}))\cos (\phi ) + \sin (\omega (t - \tfrac{r}{c}))\sin (\phi )]\hat{\theta }} \right. \hfill \\ \left. { - [\cos (\omega (t - \tfrac{r}{c}))\sin (\phi ) + \sin (\omega (t - \tfrac{r}{c}))\cos (\phi )]\hat{\phi }} \right\} \hfill \\ \end{gathered}$$

6 $$\begin{gathered} \mathop{B}\limits^{\rightharpoonup} = \frac{{\mu_{0} p_{0} \omega }}{4\pi rc}\left\{ {\left[ {\cos (\omega (t - \tfrac{r}{c}))\sin (\phi ) + \sin (\omega (t - \tfrac{r}{c}))\cos (\phi )} \right]\hat{\theta }} \right. \hfill \\ \left. { + \cos (\theta )\left[ {\cos (\omega (t - \tfrac{r}{c}))\cos (\phi ) + \sin (\omega (t - \tfrac{r}{c}))\sin (\phi )} \right]\hat{\phi }} \right\} \hfill \\ \end{gathered}$$

where *p*_*0*_ is the dipole moment magnitude, (*p*_*0*_ = *q·r*_*arc*_), *ω* is the charge’s angular velocity (*ω* = 2π*v*_*sat*_*/r*_*arc*_), *µ*_*0*_ is the vacuum permeability, *t* is the time parameter; *r*, *θ*, and *ϕ* are the respective radial, altitude, and azimuth coordinates with corresponding unit vectors $\hat{r}$, $\hat{\theta }$, and $\hat{\phi }$.

Unlike the rotating electric dipole problem, it is not possible to make fully circular trajectories while maintaining maximal separation between source and drain terminals (Fig. 1), hence the semicircular pattern was chosen for the device geometry. The solutions to the classic problem must then be modified by multiplying by a window function [*H*(*t*)* – H*(*t – T/2*)], where *H(t)* is the Heaviside step function ^33^. A Fourier transform of the modified classical solution is calculated over the valid times of 0 to *T*/2 where *T* is the orbital period in the classic problem.

A dimensionless and reparametrized expression for the Fourier transformed fields is given in Eq. (7) where the dimensionless frequency, x, is the ratio of the frequency parameter ω to the charge’s angular velocity *ω*_*0*_. Given the equivalence of the electric and magnetic fields by multiplication of an orthogonal unit vector and a factor of the speed of light, depending on the unit system— only the electric field is shown:

7 $$\mathop{E}\limits^{\rightharpoonup} (x,\phi ,\theta ) = \frac{{(1 + e^{ - i\pi x} )(\cos \phi - ix\sin \phi )\hat{\phi } + \cos \theta (1 + e^{ - i\pi x} )(\cos \phi + ix\sin \phi )}}{{x^{2} - 1}}\hat{\theta }.$$

An expression for dimensionless Fourier transformed Poynting vector $\overrightarrow{S}=\overrightarrow{E}\times \overrightarrow{H}$ can be written as follows:

8 $$\mathop{S}\limits^{\rightharpoonup} = \frac{{2(1 + \cos (\pi x))[x^{2} (1 - \cos^{2} \phi \sin^{2} \theta ) + 1 - \sin^{2} \phi \sin^{2} \theta ]}}{{(x^{2} - 1)^{2} }}\hat{r}.$$

**Figure 2:**
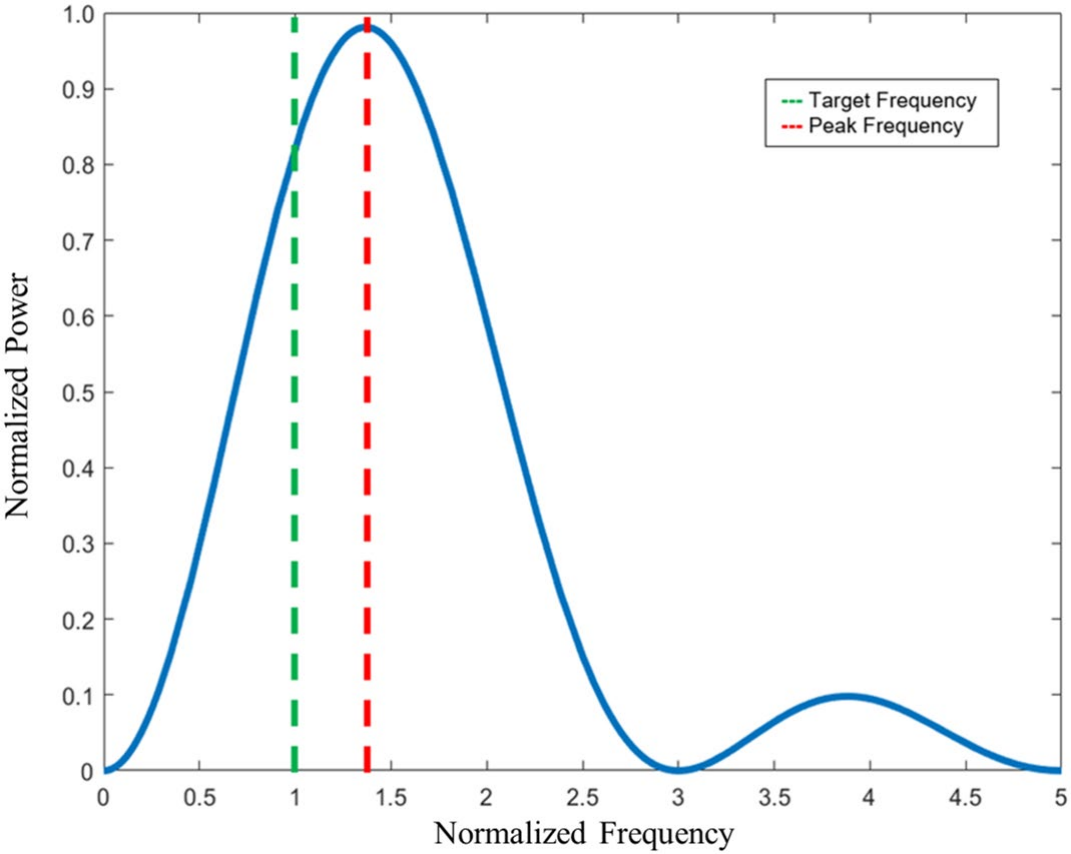
Normalized simulated transient rotating electric dipole spectrum. Calculated frequency spectrum for transient rotating dipole lasting ½ of a full orbital period. The designed target frequency (green) is recovered in the transient dipole model. In addition, the peak emission occurs at a normalized frequency at ~ 1.36 (red). A secondary peak occurs at ~ 3.6.

The truncated geometry results in an emissions continuum in which the first peak spans 3 times the orbital velocity with peak power output occurring at a frequency ~ 1.36 times the orbital velocity (Fig. 2). Provided that the non-equilibrium condition is satisfied, this frequency distribution is only a function of design geometry ^34^ which suggests that such a device can also behave as a band converter to emit frequencies higher or lower than the input frequency.

**Figure 3:**
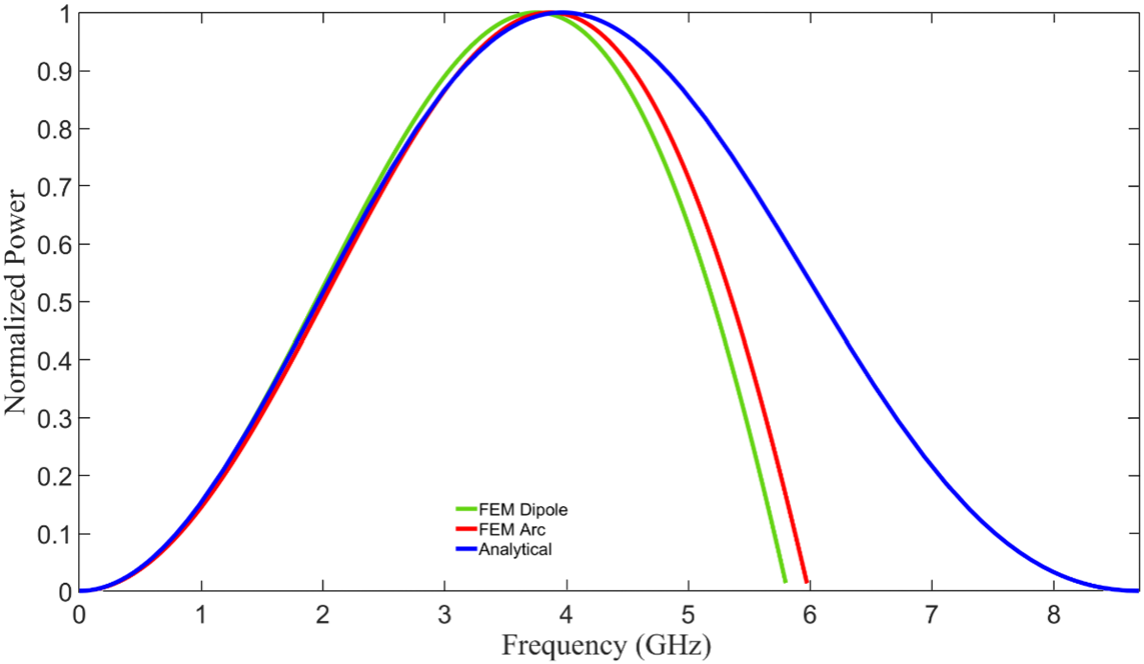
Simulated 10 µm arc radius spectrum. Simulated frequency spectrum for the 10 µm arc radius design using the measured saturation velocity of 1.87 × 10^7^ cm/s using the analytical model described here and FEM models described in Planillo et al.^15^.

The graphene semicircular arc geometries for this experiment were designed with a 10 µm radius and 10 µm width as this geometry can be readily fabricated with conventional photolithography methods. Initial characterization of commercially obtained graphene on hBN/SiO_2_/Si substrates^35^ shows that the measured saturation velocity is 1.87 × 10^7^ cm/s ^28,29,36,37^. With this saturation velocity, the arcs are expected to emit at a target frequency of 2.9 GHz at a power of 3.96 nW as given by Eqs. (3) and (4). The full frequency distribution for this design is shown in Fig. 3. Finite element models were developed to simulate this structure in which transient stimuli were applied the structure and compared to the analytical solution and equivalent rotating electric dipole. A detailed description of the models in COMSOL Multiphysics can be found in Planillo et al.^34^. The results show peak emissions between 3.74 and 3.94 GHz. Instrumentation for this range of frequencies is mature and a successful demonstration would serve as a proof-of-concept. Higher frequencies can then be achieved by scaling via a reduction in the arc radius.

**Figure 4:**
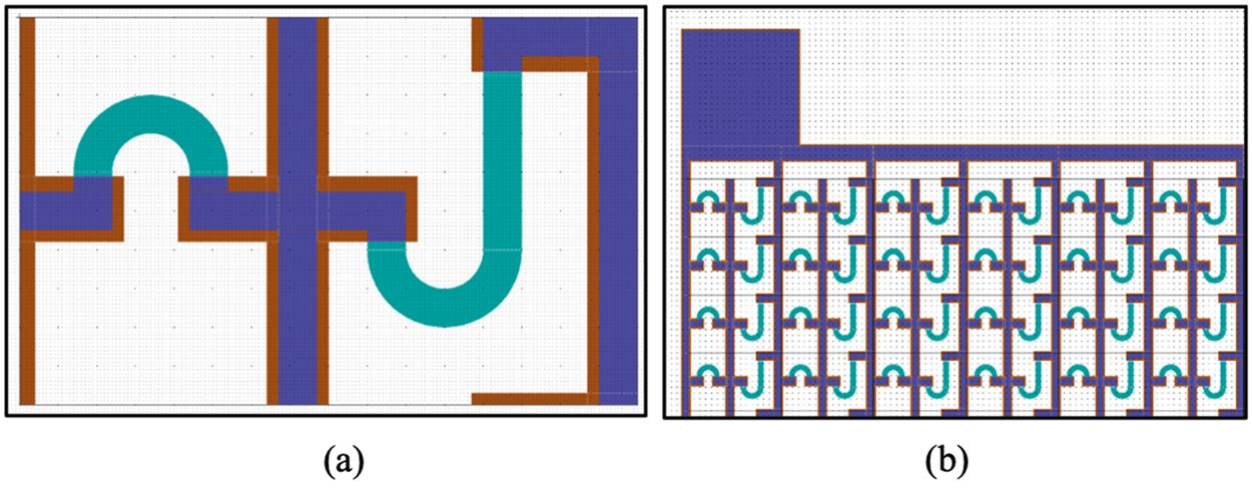
Graphene arcs layout. (**a**) Array unit cell consisting of a semicircular arc and an inverted arc with π phase delay to make a “J” shape. (**b**) Top right corner of an array column.

In order to obtain measurable power levels of cyclotron emissions, arrays of arcs can be used. The intent of this design is to large arrays on the usable area of the 4’’ wafer. An array of the 10 µm radius arcs in a repeated unit cell configuration in Fig. 4a. A 20 µm wide interconnect of Cr/Au encapsulates the graphene except for the active radiating areas. To achieve large scale arrays, the resistivity properties of Au (0.44 Ω/sq ^38^) are more desirable than that of graphene (450 Ω/sq). Encapsulation also mitigates high contact resistance common with metal/graphene interfaces ^39–41^. The active radiating areas of the unit cell (Fig. 4a) consists of a semicircular arc with an adjacent “J” shaped arc. By reversing the direction of the adjacent arc, the central metallic interconnect can be shared while preserving the rotation for both arcs. An additional straight graphene patch with length equivalent to *πr*_*arc*_ is also added to form a “J” shaped arc to introduce a π phase delay. The introduction of the phase delay should prevent simultaneous emission from both arcs which may result in destructive interference.

The unit cell—measuring 160 µm × 100 µm—is repeated to make a 129 × 70 unit stack, partially shown in Figure 4b. In total, the designed wafer layout contains 288,960 arcs.

As part of the experimental process, a twin wafer was fabricated using exactly the same process. It was then subjected to an additional O_2_ plasma etch for 20 s. This would remove the exposed graphene in the active radiating areas and leave only the metallic network. A comparison between the emissions of the two wafers will distinguish which emissions are due to the metallic network and which emissions are due to the graphene arcs.

**Figure 5:**
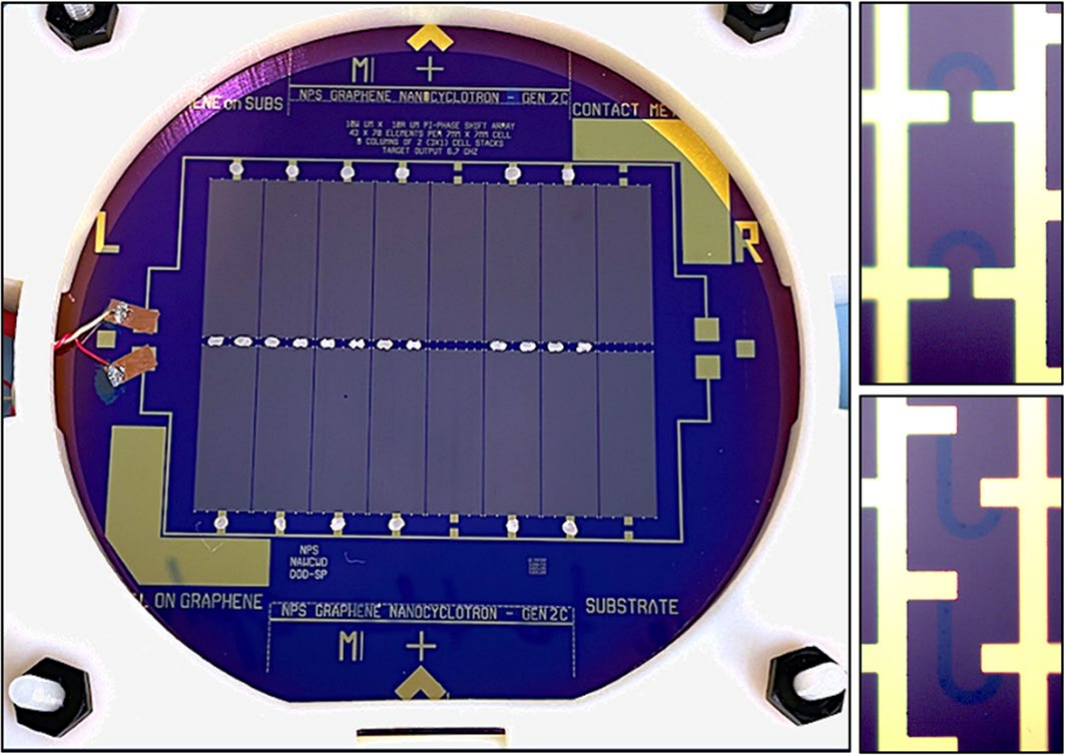
Packaged Wafer. Graphene features showing on the right. Post-fabrication packaging of the 4’’ wafer. Electrical contact between the columns and the bus bars are made via conductive silver paste. A coaxial cable is soldered to the large contact pads on the bus bars.

The fabricated wafers were terminated with short coax cables and packaged in custom designed 3D printed plastic enclosures as shown in Fig. 5.

## Cyclotron emission measurements

**Figure 6:**
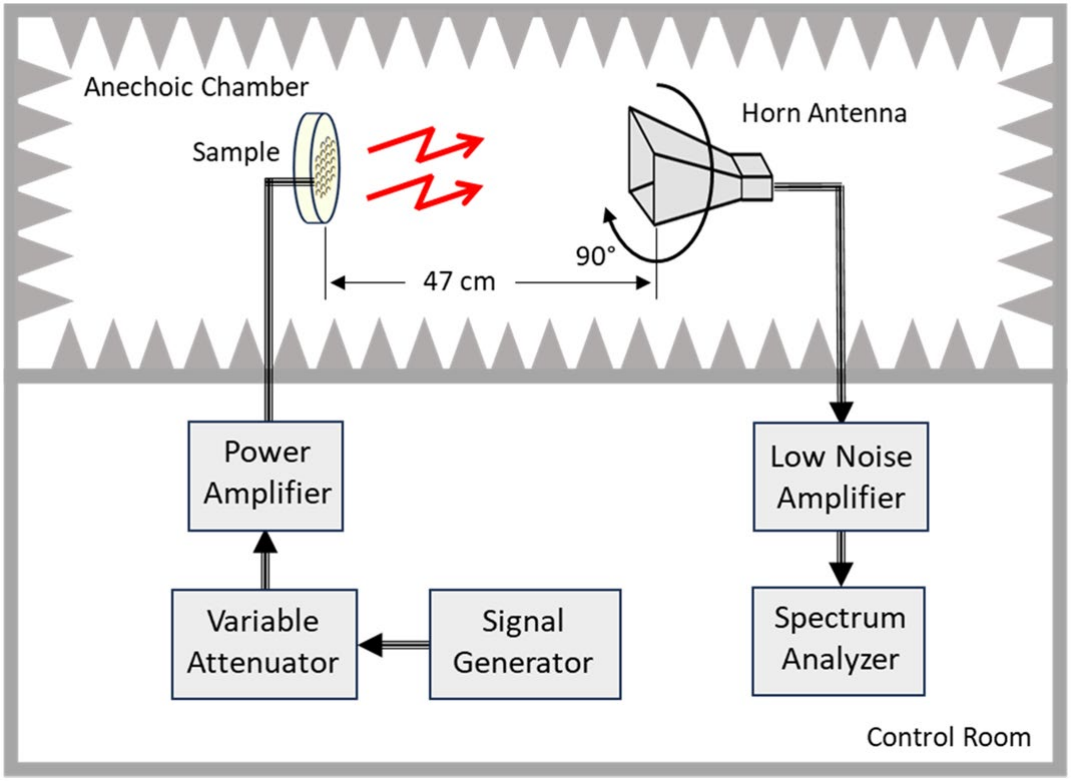
Cyclotron emission measurement diagram. Stimulus is provided by a signal generator whose output is routed through a variable attenuator and a power amplifier before connecting to the sample. Emissions from the sample are incident on a horn antenna which feeds into a low noise amplifier. The received amplified signal is then fed into the spectrum analyzer for measurement and recording.

Cyclotron emission measurements were performed at room temperature in an anechoic chamber. The experimental setup schematic diagram is shown in Fig. 6. The samples were placed at 47 cm from the receiving horn antenna (AEL H-1498). In the resulting measurements, the vertical polarization was defined as the horn elements parallel to the length of the wafer’s columns. The horizontal polarization was obtained by rotating the sample 90 degrees. A 10-foot coaxial cable connects the antenna to a 20 dB low noise amplifier (RF Bay LNA-8G) followed by a 3-foot coaxial cable connected to an Agilent E4407B spectrum analyzer.

Stimulus was provided by an HP 8350B signal generator, connected by a 3-foot coaxial cable to a variable attenuator (−50 dB to 0 dB, 10 dB step). Another 3-foot cable connects the variable attenuator to a power amplifier (QPJ-02183050). Lastly, a 6-foot cable from the power amplifier connects to the samples under test (Fig. 6). The excitation frequencies were 1.73 GHz and 10.16 GHz, at which both cyclotron and reference samples were exhibiting the same returning loss and outside of the predicted cyclotron emission band (Fig. 10).

**Figure 7:**
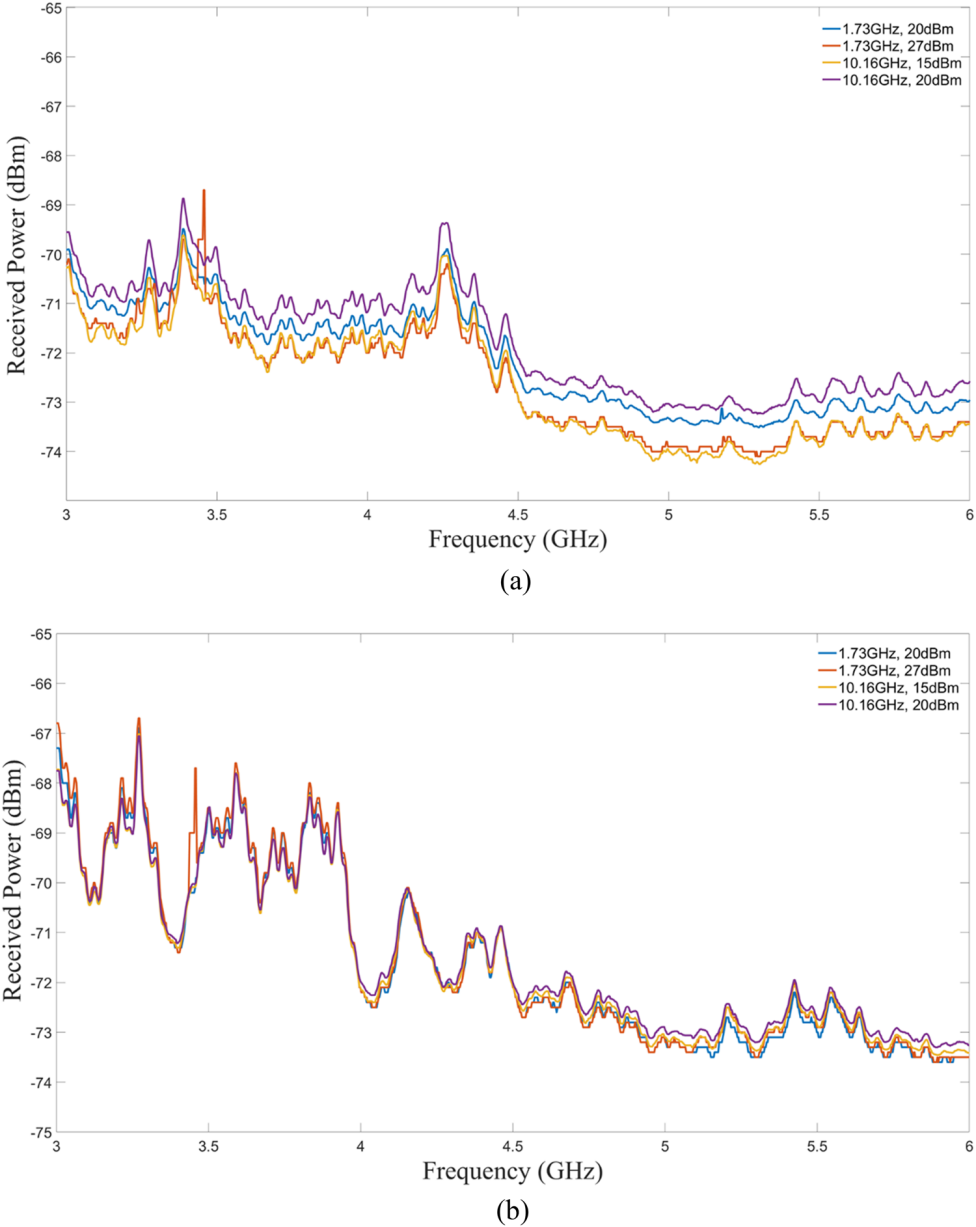
Emission spectra of graphene cyclotron array wafer. Selected measurements of the emissions from the sample with graphene present with 1.73 GHz and 10.16 GHz stimulus applied at 20 dBm and 27 dBm and 15 dBm and 20 dBm, respectively. (**a**) Vertical polarization, (**b**) horizontal polarization.

Stimulus was applied to both samples over a range of power levels. For the 1.73 GHz stimulus, powers of 10 dBm, 15 dBm, 20 dBm, 27 dBm, and 30 dBm were applied. For the 10.16 GHz stimulus, powers of 10 dBm, 15 dBm, and 20 dBm were applied. Data was collected over the frequency span of 3 GHz to 6 GHz and averaged over more than 8000 trials—the maximum allowable for the E4407B spectrum analyzer—for both polarization states. A summary of these measurements from the graphene sample is shown in Fig. 7. For the 1.73 GHz stimulus, the 20 dBm and 27 dBm measurements are displayed, while the 15 dBm and 20 dBm measurements are displayed for the 10.16 GHz stimulus. Harmonics from the respective stimuli were subtracted for clarity and further data analysis.

In Fig. 7, it is observed that all traces are within 1 dB of each other. Emissions from the horizontal polarization were more tightly grouped than the vertical polarization indicating that the emissions were mostly due to the metallic network since their predominant direction coincides with the antenna polarization. This observation was consistent with the S_21_ measurements for the horizontal polarization (Fig. 12) where the metallic network was the dominant emissions source.

**Figure 8:**
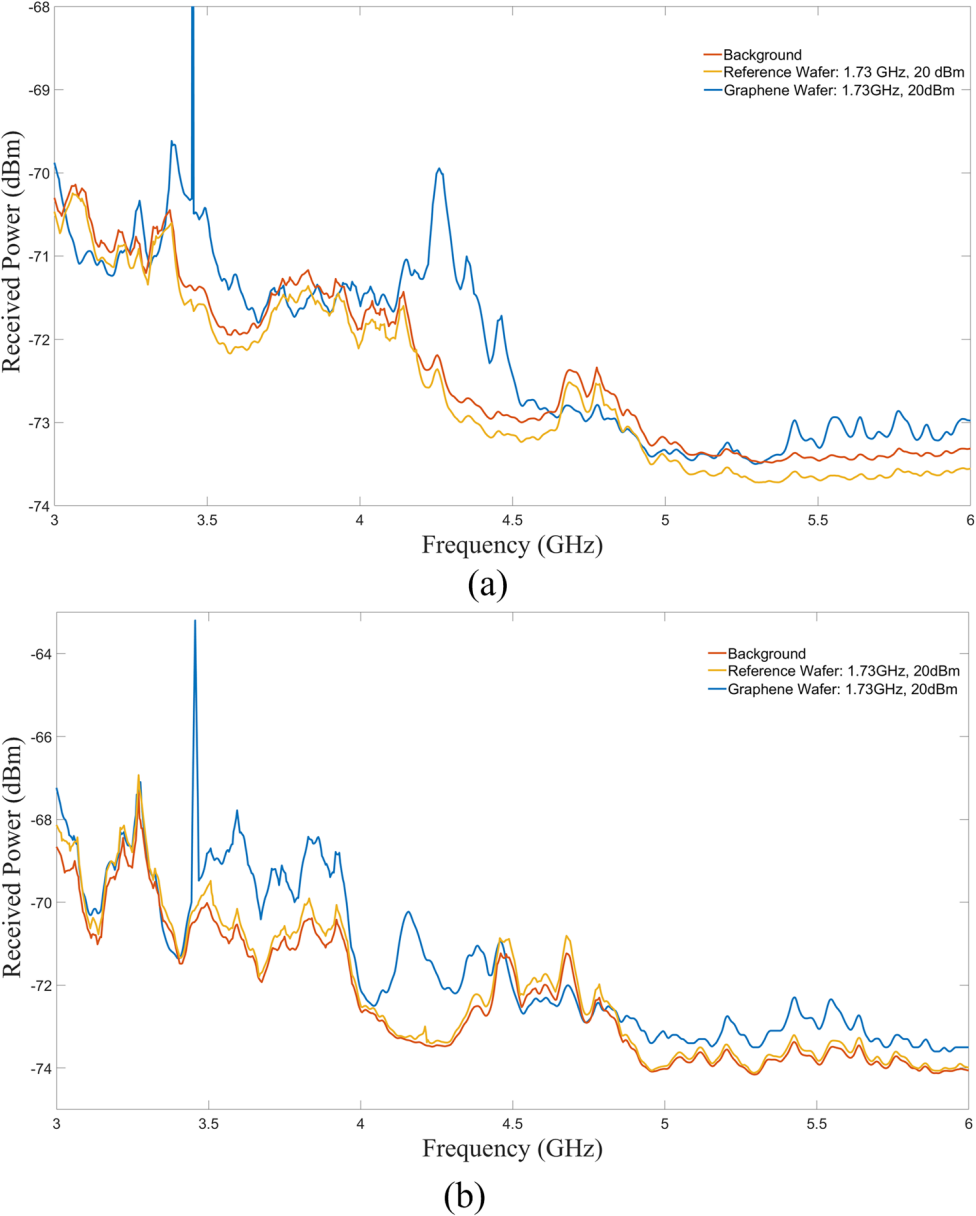
Emission measurements comparison with the graphene wafer, reference wafer, and background. (**a**) Vertical polarization, (**b**) horizontal polarization.

The emissions seem to be independent of the input stimulus frequency and power. If these emissions are due to the cyclotron radiation, it is likely that only the same number of elements are contributing to the radiation. Several measurements were performed with the graphene and reference wafers stimulated with different power levels as well as without any stimulus to obtain the noise floor of the setup. In most cases the results were similar to the one shown in Fig. 8, for a specific case where the stimulus frequency was 1.73 GHz at 20 dBm power.

## Discussion

**Figure 9:**
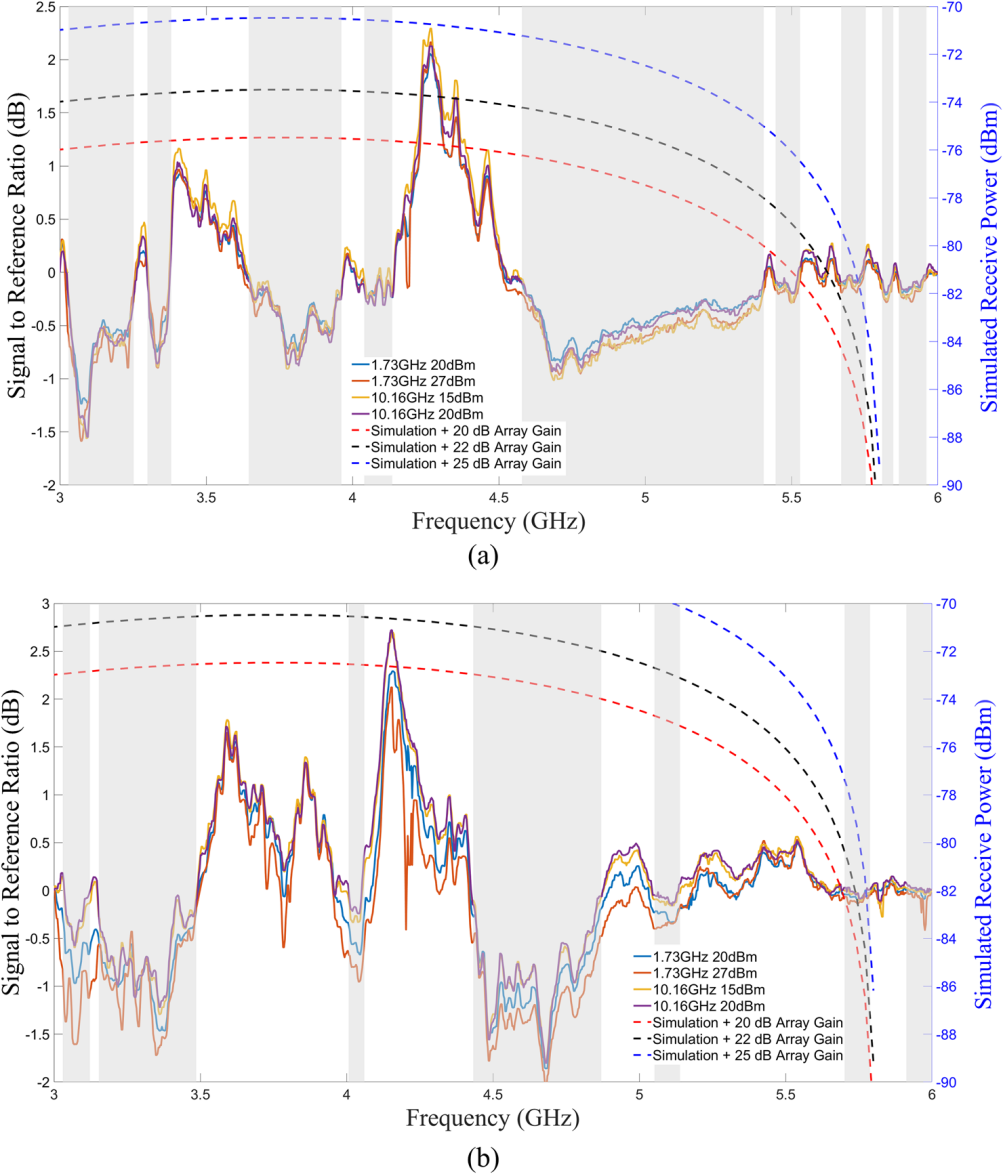
Measurement and simulation comparison. Measured data SRR (left axis) compared to simulation data (right axis). Assuming coherent emission, an array gain of 22 dB is required to meet the measured data. Notches due to band gap are highlighted in gray. (**a**) Vertical polarization. (**b**) Horizontal polarization.

Analysis was performed in the form of signal-to-reference ratio (SRR) to isolate the cyclotron emissions from the metallic network emissions. The SRR was obtained by subtracting the measured emissions from the graphene wafer from the emissions from the reference wafer. Any net positive readings would be due to the presence of graphene and subsequent cyclotron style emissions. The SRR curves, shown in Fig. 9, indicate that a net emission in the predicted range of 3 GHz to 6 GHz was detected. The SRR peaked at 4.15 GHz (2.7 dB) for the horizontal polarization. For the vertical polarization, the SRR peaked at 4.26 GHz (2.3 dB). Within the SRR analysis, there were instances of negative values. At these regions, the measured emissions from the metallic network exceeded the emissions from the graphene arcs. Additionally, harmonics from the stimulus signal were removed from the data.

To better understand the emitted power and device effectiveness, after accounting for system losses (cable loss, insertion loss, amplifier gain, propagation loss) (Table 1), the effective array gain based on simulated power was plotted along with the measured data (Fig. 9). Assuming coherent emission from the array, the measured power levels were consistent with an array gain of 22 dB or 150 effective units. To arrive at this figure, a simulated receive power was estimated using the received power from the arrays of graphene arcs, shown in Fig. 8, and subtracting the loss of the system as determined by the link budget (Table 1). The gain was then estimated by using the predicted single element power from the introduction section and multiplying by an array gain factor to arrive at the measured receive power. Figure 9 gives a graphical solution in which the array gain curve is the closest to the peak measured data for both polarizations. It is clear that the 22 dB array gain curve is the closest.

**Table 1:** Link budget.

Component	Lower loss (dB)	Upper loss (dB)
Cables from PA	−3.0	−4.0
Device insertion loss	−0.5	−1.3
Free space propagation loss (3 GHz, 6 GHz)	−35.4	−41.4
Rx antenna gain	6.0	6.0
Cables from antenna to LNA	−13.0	−15.0
LNA Gain	20.0	18.0
Cables from LNA to spectrum Analyzer	−3.0	−4.0
Total (dB)	−28.9	−41.7

**Figure 10:**
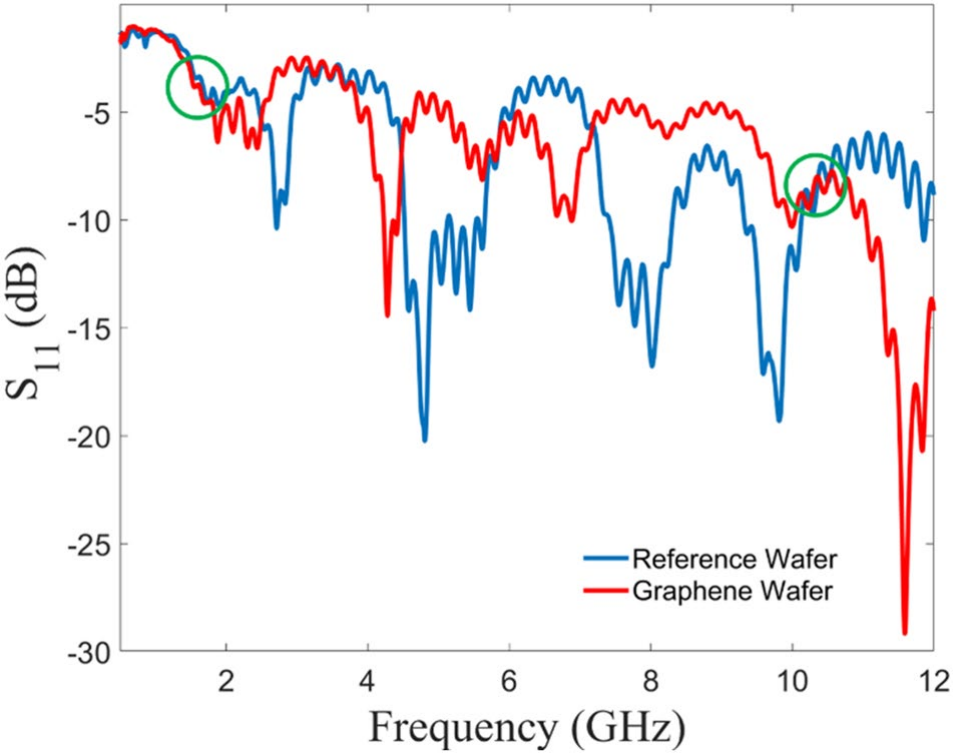
S_11_ Measurement. Scattering parameter measurement (S_11_) of the graphene (red) and reference (blue) wafers. For testing, it was necessary to find a stimulus frequency away from the target frequency where both graphs intersect (green circles): 1.73 GHz and 10.16 GHz.

The effective array gain is low given the total number of array elements. Even after factoring in manufacturing defects and contamination, at worst a 30% yield would be expected. Poor impedance matching would bring the yield even lower to 10%–15%. Since the graphene structures were precisely shaped by standard photolithography processes (relatively large structures), this low yield of coherently emitting arcs indicate that the graphene uniformity must have been compromised either during its deposition or during the structure fabrication. Further investigation is required to address this problem. Another notable aspect is that the simulation shows broadband emissions over the measured frequency range whereas the measurements exhibit band gaps (Fig. 9). The simulated estimation was performed on a unit cell basis while the measurements were performed using an array of elements. The emission gaps can be associated to the metallic network (Figs. 4b and 5) and/or the array effects of the periodically distributed graphene cyclotron elements (destructive interference). These effects are also observable in the S_11_ measurements (Fig. 10).

What is clear from the measurements is that the SRR show consistent emissions between around 3.5 to around 4.5 GHz independent on the stimulation frequency (1.73 GHz and 10.16 GHz). With graphene arcs being the only difference, the emissions are most likely to be from cyclotron radiation as predicted by the models.

Electromagnetic theory^12^, along with the authors’ previous theoretical work^34^ indicate that the emitted radiation from the devices will be circularly polarized. If the emissions were circularly polarized, one would expect to find nonzero power measurements for both polarization axes at a given frequency. In the case of perfect circular polarization, power measurements for both horizontal and vertical axes would be identical. From Fig. 9, there are instances of frequencies for which both polarizations are non-zero. The existence of non-zero power measurements for a given frequency on both axes; however, may not be sufficient to prove that the emissions are circularly polarized especially with array effects. These non-zero power measurements for both axes may be a result of superposition effects resulting from a more complicated radiation pattern. To fully characterize the polarization state, time domain measurements with a dual polarized antenna would be required to observe the rotation of the polarization vector. Such a measurement would first require taking a frequency domain measurement (this work) to identify which frequencies are emitting. This characterization is a subject of future work.

## Conclusion

Micrometric graphene arcs were fabricated to experimentally demonstrate cyclotron emission, dictated only by the graphene transport properties and geometry of the arcs. For that, two sample wafers were fabricated, one with the graphene arcs and the metallic contact lines and the other only with the contact lines to be used as a reference. Signal-to-reference ratios were obtained by subtracting the emissions of reference sample from the emissions of the sample with graphene arcs. The signal-to-reference emitted frequencies are consistent with the transient rotating dipole model and FE simulations. The power is also consistent with Larmor formulation and FE simulations. The experiments demonstrated that the emissions were independent of the input stimulus which is indicative of the cyclotron radiation mechanism and cannot be explained with theories associated with conventional ohmic conductors such as antenna theory. As such, these devices can find potential use as band converters.

The shape of the emissions spectrum differed from the simulation. This was expected since array effects were not considered in the simulations. The graphene arc array and metallic contact lines configuration were most likely creating observed bandgaps. Some of these effects can be inferred from the S_11_ measurements. The array effects on the emissions as well as uniformity and contamination of the graphene prior and after fabrication will be addressed in future work. As of this writing, we have not found any other experimental demonstration of a cyclotron emissions from graphene microstructures in the open literature.

Given the proper scaling, arrays of these devices can be THz emitters that operate either in a source driven mode via microwave stimulus, or as a photoconductive mode with laser stimulus. As these devices were manufactured with commercially available graphene/hBN wafers, these results also indicated that the manufacturing processes and quality of these stock materials can be made mature enough for further deployment. This approach may help realize cost effective access to the THz spectrum.

## Methods

The experimental phase begins with the fabrication of the graphene devices. The graphene/hBN/SiO2/Si wafers were purchased from a commercial supplier CheapTubes Inc^35^. The graphene is grown via chemical vapor deposition on Cu films^24–26^ and the wet transferred onto to the 4’’ hBN/SiO2/Si wafers. The graphene was first patterned using a 5’’ mask containing an array of semicircles. A layer of SPR-955-0.9 photoresist was spin coated onto the wafer followed by the pre-exposure bake. The wafer was then exposed to the mask pattern followed by a post exposure bake. The wafer was developed in a MicropositTM CD-26 developer solution. Upon satisfactory developing, the wafer was then subject to an O_2_ plasma reactive ion etch (RIE) to remove the unmasked graphene. After the RIE, the wafer was then subject to an acetone/isopropanol rinse to remove the remaining photoresist. The metallic layer was pattern in a similar process. Following development, a 5 nm Cr adhesion layer was deposited followed by a 50 nm Au layer—both using a sputtering process (Angstrom NEXDEP). The excess metal and photoresist were then removed via a liftoff process in an acetone bath.

The devices were characterized to verify the survivability of the graphene to the fabrication processes using Raman microscopy. The electric characteristics of the graphene devices were measured using a Hall effect apparatus (Leybold 58681) and a parametric analyzer (Keysight B1500A). The wafer with the graphene arcs exhibited total resistance of 7.75 kΩ whereas the reference wafer showed open circuit readings thus verifying that the graphene structures were indeed removed. The methods and procedures are detailed in Planillo et al.^36^.

Preliminary RF characterization was conducted in the form of a scattering parameter (S_11_) measurement—also known as return loss—using an Agilent N5222A vector network analyzer (VNA). This was done applying a fixed input power to the sample over a span of frequencies and measuring the returned power from the same device port.

The results are shown in Fig. 10. To demonstrate the cyclotron radiation process, the wafer requires stimulation away from the target frequency. To make a fair comparison, both the reference and graphene wafer were stimulated at frequencies where their return losses were the same, as this assumes that both the reference and graphene wafers will receive the same power. These frequencies were determined to be 1.73 GHz and 10.24 GHz with S_11_s of −4.44 dB and −8.96 dB, respectively (Fig. 10—green circles). These S_11_s fall below -10 dBm (10% of incident power reflected)^42,43^. A matching network can be implemented to improve the return loss, however, the matching network may inadvertently bias the emissions that are being investigated towards the matching frequency, compromising the measurements.

**Figure 11:**
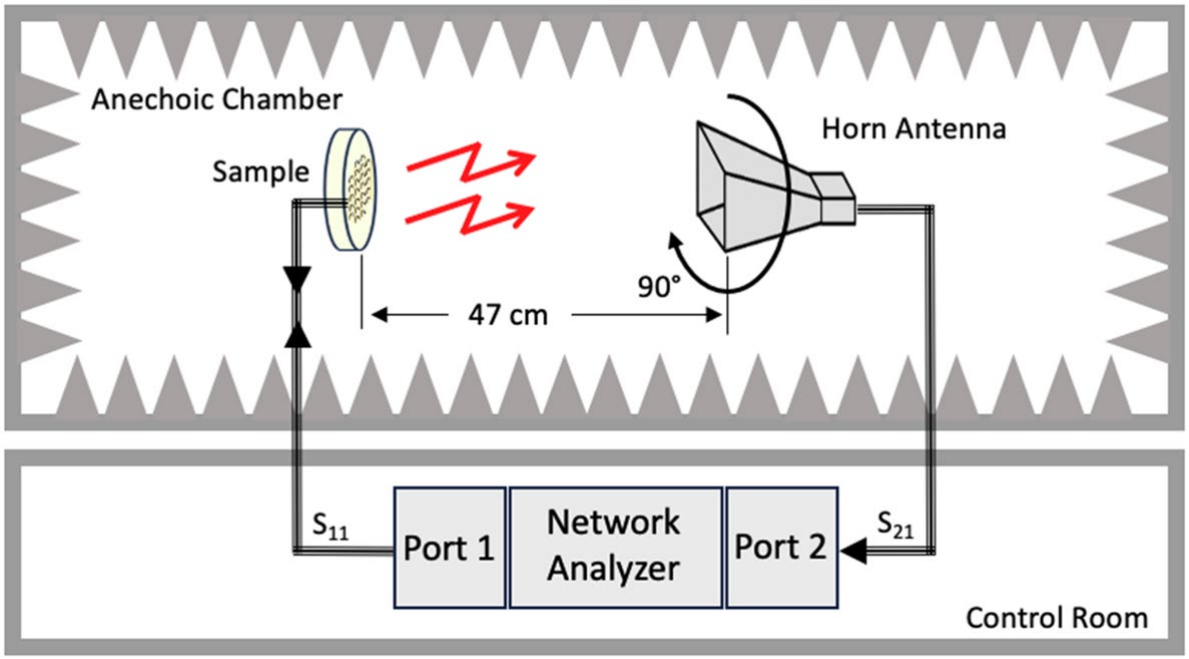
S_21_ Experimental setup to measure the S parameters. (**a**) S_21_ characterization begins with stimulus applied from VNA port 1 to the sample. Emissions propagate through free space which are received by a horn antenna connected to VNA port 2. (**b**) A fabricated sample undergoing an S_21_ measurement.

The insertion loss (S_21_) was measured using an Agilent FieldFox N9918A VNA. This was performed applying a fixed input power, from port 1, to the sample over a span of frequencies and measures the transmitted power, after propagating through free space, via horn antenna (AS-48461) connected at port 2 (Fig. 11). A separation distance of 47 cm from the sample to the receive horn antenna was chosen as this is the minimum distance to achieve the far field condition at the highest expected frequency of 6 GHz. The S_21_ measurements served as the first indication if the sample radiates and can reveal any polarization dependencies of the emissions. Horizontal and vertical polarizations were obtained by rotating the horn antenna by 90°, with the vertical polarization defined as the horn elements parallel to the length of the wafer’s columns. The S_21_ measurements were performed at room temperature in an anechoic chamber.

**Figure 12:**
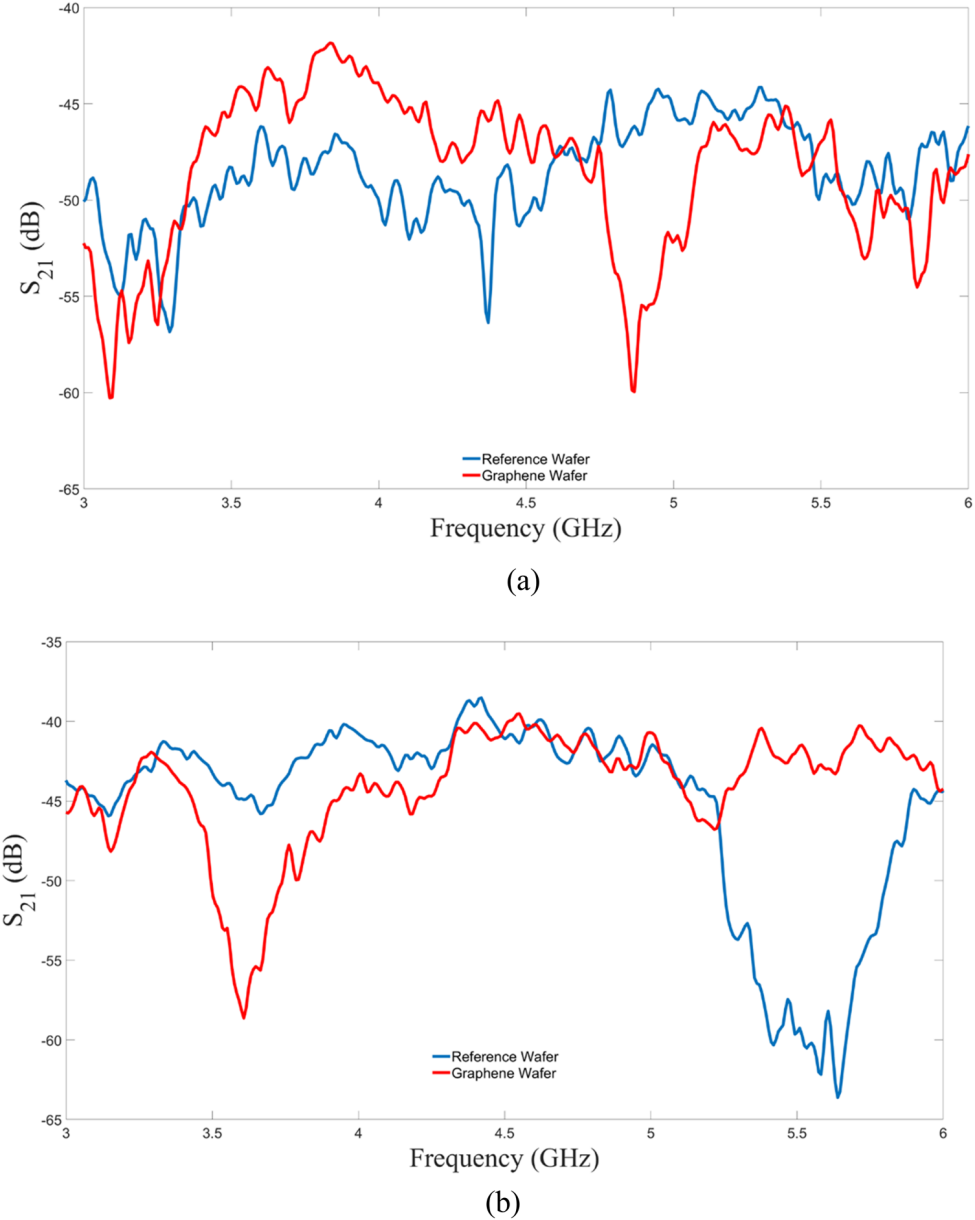
S_21_ measurement. (**a**) Vertical polarization, (**b**) horizontal polarization.

As it can be observed in Fig. 12, for both polarizations, both wafers radiated across the span of 3 GHz to 6 GHz above the instrument’s detection threshold of -65 dB. For the vertical polarization, the graphene wafer transmitted more power than the reference wafer from 3.4 to 4.5 GHz. For the horizontal polarization, the opposite was observed in which the reference wafer transmitted more power over the same range. The S_21_ results assisted in the expectations and interpretations of the cyclotron emission measurements.

## Data Availability

The datasets used and/or analysed during the current study available from the corresponding author on reasonable request.
